# The Impact of Flavonoid Supplementation on Serum Oxidative Stress Levels Measured via D-ROMs Test in the General Population: The PREVES-FLAVON Retrospective Observational Study

**DOI:** 10.3390/nu16193302

**Published:** 2024-09-29

**Authors:** Giuseppe Di Lorenzo, Antonio Verde, Luca Scafuri, Ferdinando Costabile, Vincenza Caputo, Rossella Di Trolio, Oriana Strianese, Vittorino Montanaro, Felice Crocetto, Francesco Del Giudice, Raffaele Baio, Antonio Tufano, Paolo Verze, Alessia Nunzia Calabrese, Carlo Buonerba

**Affiliations:** 1 Oncology Unit, “Andrea Tortora” Hospital, ASL Salerno, 84016 Pagani, Italy; direttoreuocpagani@gmail.com (G.D.L.); antonioverde93@gmail.com (A.V.); ferdicostabile@gmail.com (F.C.); xt.strianeseo@aslsalerno.it (O.S.); alessianunziacalabrese@gmail.com (A.N.C.); 2 Associazione O.R.A. ETS-Oncology Research Assistance, 84134 Salerno, Italy; carbuone@hotmail.com; 3 UniCamillus—Saint Camillus International University of Health Sciences, 00131 Rome, Italy; 4 Oncology Unit, “Luigi Curto” Hospital, ASL Salerno, 84035 Polla, Italy; vincenza.caputo.92@gmail.com; 5 Unit of Melanoma, Cancer Immunotherapy and Development Therapeutics, Istituto Nazionale Tumori Istituto di Ricovero e Cura a Carattere Scientifico Fondazione G. Pascale, 80131 Naples, Italy; r.ditrolio@istitutotumori.na.it; 6 Urology Department, “San Leonardo” Hospital—ASL Napoli 3 Sud., Castellammare di Stabia, 80053 Naples, Italy; vittorino.montanaro@alice.it; 7 Urology Unit, Department of Neurosciences, Reproductive Sciences and Odontostomatology, University of Naples “Federico II”, 80133 Naples, Italy; felice.crocetto@gmail.com; 8 Department of Maternal Infant and Urologic Sciences, “Sapienza” University of Rome, Policlinico Umberto I Hospital, 00161 Rome, Italy; francesco.delgiudice@uniroma1.it; 9 Department of Urology, Umberto I, Nocera Inferiore, 84014 Salerno, Italy; dott.rbaio@gmail.com; 10 Unit of Urology, Istituto Nazionale Tumori IRCCS Fondazione G. Pascale, 80131 Naples, Italy; antonio.tufano91@gmail.com; 11 Department of Medicine, Surgery and Dentistry, “Scuola Medica Salernitana”, University of Salerno, 84081 Baronissi, Italy; pverze@unisa.it

**Keywords:** oxidative stress, flavonoid supplementation, primary prevention, d-ROMs, glucose, blood pressure

## Abstract

Background: Oxidative stress has emerged as a key contributor to numerous NCDs (non-communicable diseases), including cardiovascular diseases, cancer, and diabetes. This study aims to explore the potential of targeted interventions to mitigate oxidative stress as part of a primary prevention strategy. Methods: The study included 32 healthy participants (11 men, 21 women) aged 45–65 who completed both the initial and follow-up assessments of the Healthy Days Initiative, a community-based wellness program organized by the non-profit Associazione O.R.A. ETS. Through blood analysis, vital sign assessment, lifestyle questionnaires, and individualized recommendations, participants received guidance on improving their health and reducing disease risk. The initiative also offered the opportunity for participants to consume a flavonoid supplement containing quercitrin, rutin, and hesperidin, with the goal of reducing oxidative stress. Participants who opted for supplementation were instructed to take 1–2 tablets daily for two weeks. Data collected included demographic information, anthropometric measurements, vital signs, dietary and lifestyle habits, medical history, WHO-5 Well-Being Index scores, and blood parameters. Results: Significant reductions were observed in glucose levels (from 82 to 74.5 mg/dL), reactive oxygen metabolites (d-ROMs) (from 394.5 to 365.5 U.CARR), and systolic blood pressure (from 133 to 122 mmHg) after the two-week flavonoid intervention. Most participants (26/31) reported no side effects, and the majority (30/31) expressed a willingness to continue using a product combination of quercitrin, rutin, and hesperidin or a similar product long-term. Conclusions: While limited in scope and duration, the PREVES-FLAVON study contributes valuable insights to the growing body of evidence suggesting that flavonoid supplementation may play a significant role in reducing risk factors associated with NCDs in primary prevention settings. By targeting novel risk factors such as oxidative stress, this intervention holds promise for mitigating the global burden of NCDs and promoting healthy aging.

## 1. Introduction

Worldwide, the epidemiological landscape is dominated by non-communicable diseases (NCDs), imposing a substantial toll on global health. Alarmingly, 17 million individuals die from NCDs before the age of 70, with 86% of these premature deaths occurring in low- and middle-income countries (World Health Organization, 2023) [1]. Several modifiable risk factors contribute to this burden, including tobacco use, physical inactivity, harmful alcohol consumption, unhealthy diets, and air pollution. Metabolic risk factors, such as raised blood pressure, overweight/obesity, hyperglycemia, and hyperlipidemia, also play a significant role, while environmental factors, particularly air pollution, are a major contributor [2]. The economic implications are profound, encompassing direct medical costs, indirect productivity losses, and intangible societal impacts. However, mounting evidence suggests that proactive prevention strategies can yield substantial financial gains by reducing disease prevalence and associated healthcare expenditures [3].

Primary prevention, the proactive approach to averting disease onset by mitigating risk factors, emerges as a cornerstone in the quest for prolonged health and well-being, especially for individuals without existing diseases or with minimal health burdens [4]. Primary prevention of major NCDs involves a multifaceted approach, with lifestyle modifications playing a central role. Primary prevention of cardiovascular diseases emphasizes healthy lifestyle choices throughout life, a team-based care model considering individual social determinants of health, a diet rich in vegetables, fruits, nuts, whole grains, lean proteins, and fish, regular physical activity, tobacco cessation, and judicious use of medications [5]. Prevention or delay of type 2 diabetes hinges on lifestyle behavior changes, particularly weight loss and increased physical activity, with various eating patterns like Mediterranean-style, low-carbohydrate, vegetarian, plant-based, and DASH diets being considered, along with pharmacological interventions like metformin for high-risk individuals [6]. For cancer primary prevention, the World Cancer Research Fund (WCRF), the American Cancer Society (ACS), and the European Code Against Cancer recommend maintaining a healthy weight, limiting calorie-dense foods and sugary drinks, focusing on a diet rich in vegetables, fruits, whole grains, and legumes, reducing salt and red meat intake, avoiding processed meats, and limiting alcohol consumption [7,8].

Beyond implementing established lifestyle modifications, a promising avenue for intervention in both clinical practice and research lies in addressing novel risk factors. Oxidative stress, a recognized contributor to numerous non-communicable diseases, including cardiovascular events, cancer, and diabetes, presents a prime target for such interventions. Emerging clinical approaches aimed at mitigating oxidative stress levels, such as dietary interventions incorporating supplements including selected flavonoids [9,10] or specific dietary regimens, could offer innovative strategies for primary prevention. In this regard, it is noteworthy that cocoa extract supplementation, rich in flavanols (a subclass of flavonoids), was associated with a 27% reduction in cardiovascular death in a large-scale randomized controlled trial involving 21,442 US adults [11].

The Healthy Days Initiative, held on 26 May and 9 June in private offices, focused on personalized wellness and disease prevention directed at individuals self-considered to be healthy aged 45–65. Organized by the non-profit research organization Associazione O.R.A. ETS (www.oncologiaora.it (accessed on 10 August 2024)), the initiative involved blood analysis, vital sign assessment, a lifestyle questionnaire, and personalized recommendations on diet, exercise, and flavonoid supplementation.

Flavonoid supplementation was freely provided using a registered product (WaisPharma, Milan, Italy), with the recommended dosage of 1 or 2 tablets per day, aligning with Italian Ministry of Health guidelines. This approach aimed to empower participants to make informed decisions about supplementation, considering the extensive evidence supporting the potential benefits of flavonoids, following individual discussions and under medical guidance. The crucial role of a healthy diet was underscored, emphasizing that nutritional supplements should enhance, not substitute, a balanced lifestyle. This strategy reflects the organizers’ perspective that flavonoid supplementation, when used responsibly and with appropriate information, can be a valuable tool for promoting health.

The retrospective observational PREVES-FLAVON study analyzed data collected during the Healthy Days Initiative, primarily focusing on assessing changes in D-ROMS levels (a marker of oxidative stress) following two weeks of flavonoid consumption. Given the established association between elevated D-ROMS and an increased risk of cardiovascular and other diseases, our study results may contribute to the development of effective, non-invasive, and cost-efficient strategies to mitigate this known risk factor within primary prevention efforts. Furthermore, utilizing D-ROMS as a surrogate endpoint could offer a readily available and practical biomarker to assess the efficacy of various preventive interventions.

## 2. Patients and Methods

### 2.1. The Healthy Days Initiative

The Healthy Days Initiative, hosted on 26 May and 9 June 2024 in private offices generously provided by the Fondazione Scoppa, aimed to promote personalized well-being and disease prevention for healthy citizens aged 45–65 with no specific health concerns. This initiative, held outside of hospital or clinical settings, was advertised to the public and organized by Associazione O.R.A. ETS, a non-profit research organization focused on preventing non-communicable diseases. The initiative aimed to recruit 50 participants.

On 26 May 2024, participants were greeted by a trained hostess and a medical doctor (CB), who explained the day’s activities and obtained informed consent before proceeding. Vital signs (blood pressure, oxygen saturation, weight, and height) were measured by a trained nurse, and blood samples were collected for various tests (blood count, glucose, creatinine, GOT, GPT, and D-ROMS). A qualified biologist then conducted a structured interview, gathering participants’ medical history and lifestyle information, including adherence to the Mediterranean diet, common disease history, and a 5-question WHO well-being assessment. Following the questionnaire, a specialist doctor (CB) evaluated individual risk factors and provided personalized advice on disease prevention, emphasizing lifestyle modifications and adherence to the Mediterranean diet. Participants were encouraged to make dietary changes, increase physical activity, and quit smoking. Additionally, the doctor discussed the potential benefits of flavonoid supplementation based on clinical trials, experience, and expert opinion, highlighting their potential to support physiological functions rather than cure or prevent diseases. Those interested in flavonoid supplementation and who provided consent received a dietary supplement containing flavonoids (150 mg quercitrin, 150 mg rutin, and 200 mg hesperidin per tablet), provided by Associazione ORA ETS as part of the Initiative. Participants were instructed to take 1–2 tablets daily and were informed about potential effects based on registered claims and scientific data, such as increased energy and improved metabolism/microcirculation. Finally, an oncologist (FC or VC) discussed age-appropriate oncological screenings with the participants.

After 14 days, participants returned for a follow-up visit with CB, where they reviewed their blood test results, assessed lifestyle changes, and discussed the preliminary effectiveness of the interventions. Additionally, a qualified biologist conducted a structured interview specifically focused on any adverse events or subjective effects that participants might associate with the flavonoid supplement (Table 1). Also, participants were asked if they were willing to consume the provided flavonoid supplement or a similar product for a prolonged period (>6 months). Another 14 days later, a telephone evaluation was conducted by CB to gather participants’ overall impressions and reinforce the importance of healthy lifestyles.

### 2.2. Inclusion and Exclusion Criteria

Participants eligible for this retrospective observational study included citizens aged 45–65 years old, with no specific health concerns, who had participated in the Healthy Days Initiative on both Days 1 and 2 and provided initial consent for using their anonymized data for scientific purposes. Citizens who failed to fulfill both these criteria were excluded from this retrospective observational study.

### 2.3. Retrieved Data

Data were collected by investigators using an Excel file containing no identifiable patient personal data. The following data were retrieved:

Demographic information: age and sex, collected on Day 1.

Anthropometric measurements: weight (kg), height (cm), collected on Day 1.

Vital signs: systolic blood pressure (mmHg), diastolic blood pressure (mmHg), oxygen saturation (%), heart rate (bpm), collected on Days 1 and 2.

Dietary habits: Adherence to Mediterranean Diet, assessed via the food frequency questionnaire reported by Sofi et al., collected on Days 1 and 2 [12].

Smoking habits: Current smoking status, collected on Days 1 and 2.

Alcohol consumption: Actual consumption of wine, beer, and spirits, collected on Days 1 and 2.

Medical history: history of myocardial infarction, angina pectoris, stroke, hypertension, hypercholesterolemia or hypertriglyceridemia, diabetes, liver or kidney stones, intestinal polyps, malignant tumors, gastric or duodenal ulcers, and autoimmune diseases, collected on Day 1.

WHO-5 Well-Being Index: scores on five questions related to well-being (feeling cheerful and in good spirits, calm and relaxed, active and vigorous, waking up fresh and rested, and having a daily life filled with interesting things) collected on Day 1.

Blood parameters: white blood cell count (WBC), hemoglobin (Hgb), platelet count (PLT), estimated glomerular filtration rate (eGFR), glucose, creatinine (mg/dL and μmol/L), aspartate aminotransferase (AST), alanine aminotransferase (ALT), and reactive oxygen metabolites (d-ROMs) collected on Days 1 and 2.

### 2.4. Blood Tests and Physical Parameters

During the Health Days Initiative, heart rate and O_2_ saturation were measured using a pulsosymeter (OXY4, GIMA SRL, Florence, Italy). Blood pressure was measured automatically with the PIC EASY RAPID^®^ (Pikdare S.p.A., Casnate Con Bernate, Italy) device, following the guidelines of the European Society of Hypertension (ESH). Weight and height were measured by a trained nurse (Porpora Lab SRL, Baronissi, Italy) according to standard procedures: weight on a calibrated balance beam scale with participants removing shoes and heavy clothing, and height using a stadiometer with participants standing straight and head in the Frankfort plane. Blood samples were collected by Porpora Lab SRL, processed locally, and shipped within 48 h to Synlab (Synlab Italia srl, Rovato, Italy) for analysis. A complete blood count was determined using the automated hematology analyzer Sysmex XN2000 (Sysmex Corporation, Kōbe, Japan), following standard operating procedures and quality control measures. The Atellica CH Analyzer was used to measure creatinine and glucose oxidase levels. Reactive oxygen metabolites (d-ROMs) were measured using the d-ROMs test (DIACRON INTERNATIONAL, Grosseto, Italy), with reference values of 250–300 U.CARR and intra-/inter-assay CVs of 0.3–6.6% and 0.3–5.1%, respectively. All analyses were conducted according to the manufacturer’s instructions.

### 2.5. Statistical Methods

The Wilcoxon Signed-Rank test was specifically chosen to analyze changes in paired continuous variables, such as oxidative stress levels (D-ROMS) and other blood parameters, between the initial assessment and the two-week follow-up. Furthermore, we utilized descriptive statistics, including medians, interquartile ranges, frequencies, and percentages, to provide a comprehensive summary of the study population’s characteristics and the distribution of key variables at both baseline and follow-up.

### 2.6. Ethics and Privacy Policy

All collected information was carefully stripped of any details that could identify the participants, ensuring complete anonymity. The study team adhered strictly to the regulations outlined in the General Data Protection Regulation (GDPR). This commitment to privacy meant that only individuals who had previously given explicit consent for their data to be used in research were included in the analysis. The retrospective observational study was approved by the Institutional Review Board of Associazione ORA ETS (Protocol No. CE/001/2024) and strictly adhered to the principles of the Helsinki Declaration.

## 3. Results

### 3.1. Characteristics of the Study Population

Out of the 50 individuals targeted for recruitment in the Healthy Days Initiative, 46 actually participated on either Day 1 or Day 2. All eligible participants provided informed consent for both participation and the use of their anonymized data for research. Among these, 32 participants (11 men, 21 women, median age 53) met the criteria for inclusion in this retrospective study, and 31 of them consumed the provided flavonoid supplement. The remaining individuals were excluded due to not participating on both study days. Baseline characteristics are detailed in Table 2. The median BMI was 25.8 (IQR, 24.3–28.8), and the median adherence score to the Mediterranean diet was 10 (IQR, 8–11). The baseline WHO mental wellness score had a median value of 53 (IQR, 42–72). Regarding self-reported disease history obtained through structured interviews, 6.2% (2) of participants reported a history of myocardial infarction, 31.2% (10) had hypertension, 43.8% (14) had elevated cholesterol or triglycerides, 3.1% (1) had diabetes, 9.4% (3) had kidney stones, 6.2% (2) had malignant tumors, and 21.9% (7) had autoimmune diseases (Table 3).

### 3.2. Supplements Consumption

Of the 31 individuals who reported taking the provided flavonoid supplement, 6 consumed one tablet per day, while 25 consumed two tablets. Participants taking two tablets generally followed a twice-daily schedule, morning and evening. Those taking one tablet had more flexible timing throughout the day. All participants reported consistent daily consumption without missing doses. The adherence rate was very high, with 96.9% of participants confirming they took the supplement as directed for two weeks. Among these 31 individuals, 26 (83.9%) experienced no side effects, while 5 (16.1%) reported mild and temporary adverse events. These included gastrointestinal issues in 2 individuals (6.5%), nervousness or irritability in 2 individuals (6.5%), and headaches/dizziness in 1 individual (3.2%). When asked, 30 participants (96.8%) indicated a willingness to continue taking the provided supplement or a similar product for an extended period (more than six months).

### 3.3. Hematological and Clinical Parameters

**Figure 1:**
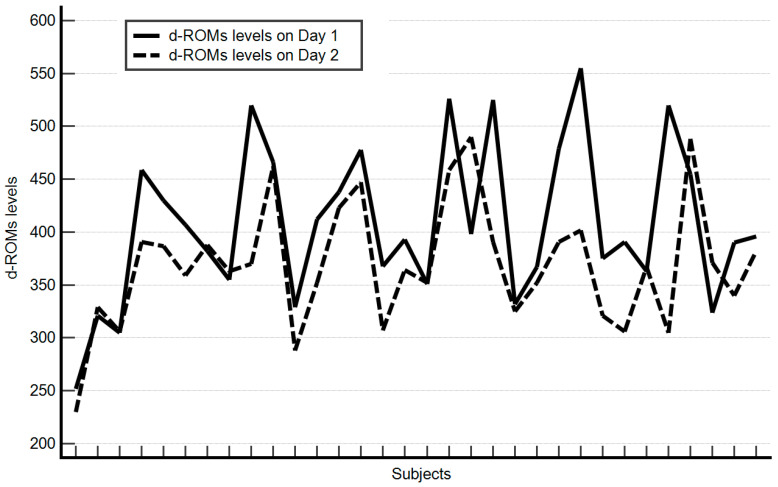
Change in Individual d-ROMS Levels from Baseline (Day 1) to Follow-up (Day 2).

At baseline, the median values of hematological parameters show hemoglobin at 14.15 g/dL and white blood cells at 6.86 × 10^3^/µL, while platelets are at 239 × 10^3^/µL. Liver function, assessed by AST and ALT enzymes, has median values of 20 U/L and 19.5 U/L, respectively. The level of oxidative stress, measured by reactive oxygen metabolites (D-ROMS), stands at 394.5 U.CARR. Cardiometabolic parameters indicated a median heart rate of 73.5 bpm, a median fasting blood sugar of 82 mg/dL, and median blood pressure readings of 133/84 mmHg (systolic/diastolic). Additionally, the median oxygen saturation was at 99%. Renal function, estimated by eGFR, shows a median value of 90. Hemoglobin, heart rate, white blood cell count, liver enzymes (GOT and GPT), platelet count, blood pressure, oxygen saturation, and estimated glomerular filtration rate did not significantly change between baseline and after 14 days. Conversely, significant reductions were observed in glucose levels (from 82 to 74.5 mg/dL, *p* = 0.00001), reactive oxygen metabolites (from 394.5 to 365.5 U.CARR, *p* = 0.0001) (Figure 1), and systolic blood pressure (from 133 to 122 mmHg, *p* = 0.00001). Follow-up assessments revealed no significant changes in adherence to the Mediterranean diet, smoking habits, or alcohol consumption during the two-week intervention period. Participants were specifically asked if they had made any changes to their medication routine, and none reported taking any additional drugs. All results are presented in Table 4.

## 4. Discussion

Oxidative stress (OS), a state of imbalance between the production of harmful free radicals and the body’s ability to counteract them with antioxidants, has emerged as a critical factor in the development and progression of numerous NCDs [13]. OS wreaks havoc on cellular components, disrupting mitochondrial function, leading to energy depletion and further ROS generation. It also damages DNA, causing mutations and genomic instability while shortening telomeres, the protective caps at the ends of chromosomes, thus accelerating cellular aging. Additionally, OS promotes lipid peroxidation, which damages cell membranes and produces harmful byproducts and modifies proteins, impairing their function [13,14,15]. Overall, these negative biological effects contribute to the pathogenesis of cancer, metabolic disorders like diabetes, and cardiovascular diseases [15]. The d-ROMs test serves as a valuable biomarker for assessing oxidative stress levels in the body. It measures the concentration of reactive oxygen metabolites (ROMs), primarily hydroperoxides, in a biological sample like blood. The test is based on the principle of Fenton’s reaction, where ROMs react with an iron-based reagent to generate free radicals [14]. Elevated d-ROMs levels have been consistently associated with a significantly heightened risk of all-cause mortality, cardiovascular disease mortality, and cancer mortality across diverse populations. A large meta-analysis [16] involving 10,622 participants from diverse European populations examined the relationship between derivatives of reactive oxygen metabolites (d-ROMs) and mortality for various causes. Of these participants, 1702 died during the follow-up period. The study found that progressively elevated d-ROMs levels (categorized as 341–400 CarrU, 401–500 CarrU, and >500 CarrU) were significantly associated with a heightened risk of all-cause mortality, with hazard ratios (HR) increasing across categories (1.27 to 4.48). Additionally, increased d-ROMs levels within these categories were associated with increased risk of cardiovascular disease (CVD) mortality (HR 1.29–5.16) and cancer mortality (HR 1.27–5.29), after adjusting for various confounding factors. Furthermore, Xuan et al. [17] conducted a large cohort study involving 2125 patients with type 2 diabetes mellitus (T2DM) and discovered that individuals in the highest tertile of d-ROMs levels exhibited a 2.10-fold increased risk of mortality compared to those in the lowest tertile et al. Finally, Gao et al. revealed that among 4345 participants, individuals within the highest tertile of d-ROM levels faced a significantly elevated risk of developing lung cancer (HR 1.90, 95% CI 1.25–2.89), colorectal cancer (HR 1.70, 95% CI 1.15–2.51), and breast cancer (HR 1.45, 95% CI 1.01–2.09), compared to those in the lowest d-ROM level tertile [18].

In the realm of primary prevention of cancer, metabolic, and cardiovascular diseases, addressing oxidative stress holds significant promise. This approach is particularly relevant given the rising global burden of NCDs and the urgent need for novel preventive strategies. Therefore, exploring innovative interventions that target oxidative stress could pave the way for novel approaches to primary prevention and ultimately contribute to a healthier population. Nutritional interventions, encompassing both dietary modifications and targeted supplementation, have demonstrated the potential to influence oxidative stress levels. While short-term dietary interventions with a Western diet did not induce changes in d-ROMs levels [19], a separate study investigating a plant-based diet, low in animal fat and rich in omega-3 fatty acids, demonstrated a significant but small reduction in d-ROMs (6%) among 104 healthy postmenopausal women [20]. A reduction in d-ROMs levels was also observed in a double-blind, randomized, placebo-controlled trial involving 380 participants aged 18–75 with a BMI ≥ 18.5 kg/m^2^ that investigated the effects of grape pomace polyphenols on d-ROMs levels. After four weeks of treatment, a significant reduction in d-ROMs was observed, decreasing from 477.08 ± 135.38 U.CARR to 313.09 ± 96.70 U.CARR (34.37% reduction, *p* < 0.0001) [21]. Numerous meta-analyses have indicated a range of potential benefits associated with flavonoid supplementation, notably including the prevention of cardiovascular and metabolic diseases [22], as well as enhancing athletic performance [23] and enhancing immune function [24]. However, studies specifically examining the impact of individual flavonoids on reducing d-ROMs levels remain limited. In a randomized, placebo-controlled, double-blind trial, forty Japanese male American football players were supplemented with either whey protein containing a particular flavonoid, that is, enzymatically modified isoquercitrin (EMIQ), or a control whey protein for four months. The group receiving EMIQ demonstrated a significant decrease in d-ROMs levels (a marker of oxidative stress) from 230.5 ± 57.2 U.CARR at baseline to 213.1 ± 50.5 U.CARR at 4 months (*p* = 0.016), with no significant change in d-ROMs levels in the control group [25]. In contrast, a formula containing 30 mg bioflavonoids from citrus, 30 mg vitamin C (as L-ascorbic acid), 10 mg coenzyme Q10, and 1 mg vitamin B-6 (as pyridoxine hydrochloride) administered for one week did not result in a significant decrease in d-ROMs levels in a small group of 14 volunteers [26].

In our retrospective study, a cohort of 32 individuals, all but one consuming a pre-defined combination of quercitrin, hesperidin, and rutin for 14 days, exhibited a significant decrease in median reactive oxygen metabolites (d-ROMs) levels from 394.5 to 365.5 (*p* = 0.0001). As shown in other studies on polyphenols [21], it is possible that a longer exposure to the flavonoid supplement could have led to even greater reductions in d-ROMs. Intriguing signals of potential biological activity from the formulation emerged, notably the decrease in median glucose levels from 82 to 74.5 mg/dL. Furthermore, the substantial drop in median systolic blood pressure from 133 to 122 mmHg is particularly noteworthy. These findings align with previous research. A systematic review and meta-analysis of 28 randomized controlled trials demonstrated that flavonoid intake significantly reduces fasting glucose and HbA1c in patients with type 2 diabetes mellitus [27]. In a small pilot study we conducted, non-diabetic patients with renal cell carcinoma receiving sunitinib therapy showed a non-significant yet clinically meaningful decrease in median fasting blood sugar levels (from 97.5 to 85 mg/dL) after taking 450–900 mg of isoquercetin orally for 10 weeks [28]. Additionally, a meta-analysis revealed that supplementation with flavonoid quercetin significantly reduced systolic blood pressure [29]. While the magnitude of the blood pressure reduction observed in our study is unexpected and likely influenced by other unaccounted-for factors, it presents an intriguing result, suggesting that the flavonoid supplement may have played a role in this positive change [30].

While our findings are promising, it is important to recognize the study’s limitations. The observational design prevents us from establishing definitive cause-and-effect relationships. Additionally, the small sample size and relatively short follow-up period may not fully capture the long-term impacts of flavonoid supplementation. It is crucial to acknowledge several limitations that necessitate a cautious interpretation of our findings: the absence of a control group, the variability in dosage (1 or 2 tablets per day), and the potential influence of unmeasured confounding factors. Furthermore, although we assessed adherence to the Mediterranean diet, a more comprehensive dietary evaluation, including detailed food and nutrient intake, would have provided a clearer picture of the participants’ dietary habits and their potential influence on the outcomes. Conversely, the study’s strengths lie in its innovative approach to primary prevention. By focusing on D-ROMs, an established but underutilized biomarker of oxidative stress, as both a risk factor and potential surrogate endpoint, we offer a proactive and targeted strategy for interventions in the general population. Moreover, our study employs a unique formulation of flavonoids (quercitrin, rutin, and hesperidin) not extensively investigated before, potentially providing new insights into the synergistic effects of these specific flavonoids on oxidative stress and associated health outcomes. The high rate of participants (30/31) expressing a willingness to continue using the flavonoid supplement or a similar product long-term suggests that it was well-tolerated and perceived to have positive effects. This subjective feedback further supports the potential role of flavonoid supplementation in promoting well-being and reducing the risk of NCDs, aligning with the existing literature on their antioxidant properties.

Although current clinical evidence does not conclusively demonstrate the superiority of any specific flavonoid or combination, several factors influenced our choice of a supplement containing quercitrin, rutin, and hesperidin. This includes the study product’s availability and adherence to the Ministry of Health’s recommended daily flavonoid dosage (maximum 1 g per day). Importantly, existing literature supports the antioxidant potential of each active ingredient in the formulation. Quercitrin, a quercetin glycoside, offers enhanced bioavailability compared to quercetin [31]. A randomized trial using a structurally similar quercetin glycoside (enzymatically modified isoquercitrin) demonstrated a reduction in D-ROMS in Japanese male American football players [25]. Furthermore, extensive in vitro data confirms the potent antioxidant activity of both hesperidin [32] and rutin [33].

While not providing definitive answers, our study contributes valuable data and insights that can inform future investigations and potentially guide the development of more targeted and effective primary prevention strategies. In this study, we focused on d-ROMs as a marker of oxidative stress. Although there are many markers of oxidative stress, such as 8-OHdG, we chose to focus on d-ROMs due to its well-established clinical utility and accessibility. The d-ROMs test is widely available and can be readily performed in routine clinical practice, making it a practical choice for assessing oxidative stress levels in the general population. Furthermore, there is a substantial body of literature that supports the association between elevated d-ROMs levels and increased risk of various diseases, underscoring its relevance as a risk factor worth addressing in primary prevention efforts.

## 5. Conclusions

In conclusion, the PREVES-FLAVON study provides preliminary evidence suggesting that a specific flavonoid supplementation, delivering 500 mg of flavonoids per tablet, may reduce oxidative stress and potentially contribute to improved glycemic control and blood pressure in healthy individuals. Notably, the PREVES-FLAVON study is the first to employ such a novel combination of flavonoids, specifically the flavonols rutin and quercitrin, alongside the flavanone hesperidin. While the relative importance of these specific flavonoids compared to others and the necessary duration of supplementation remain unknown, these findings highlight the potential value of incorporating flavonoid-rich foods or supplements into preventive strategies for major NCDs, particularly in individuals exhibiting high levels of oxidative stress as measured by the d-ROMs test. Our results provide insights into the possible mechanisms through which flavonoids may mitigate the risk of cardiometabolic diseases, and potentially even cancer, as well as the utility of the d-ROMs test for identifying at-risk individuals and assessing intervention efficacy. Future research should include a prospective placebo-controlled trial with a long follow-up period (e.g., 2–3 years or more) to evaluate the long-term impact of flavonoid supplementation on morbidity and mortality associated with NCDs. This research will help identify the most effective flavonoids and dosages and will contribute valuable data for the integration of flavonoid supplementation into broader public health initiatives aimed at NCD prevention. Ferdinando Costabile Felice Crocetto

## Figures and Tables

**Table 1:** Structured interview on reported adverse events and subjective benefits.

Subjective Benefits (Structured Interview)	Reported Adverse Events
-Strengthening of the immune system -Improvement of cognitive function -Reduction of the feeling of tiredness and heaviness in the legs -Improvement of blood circulation -Increase in energy levels -Improvement of mood and general well-being -Stress reduction -Improvement of sleep quality -Improvement of digestive function -None -Other (please specify)	-Gastrointestinal disorders (nausea, vomiting, diarrhea, constipation, abdominal pain) -Headache or dizziness -Allergic reactions (skin rash, itching, hives) -Alterations in taste or smell -Drowsiness or insomnia -Nervousness or irritability -Other side effects (please specify) -None

**Table 2:** Baseline characteristics of the study population (n = 32).

**Variable**	**Categories**	**Percentage**
Gender	Males	34.4%
	Females	65.6%
Smoker	Yes	21.9%
	No	78.1%
Consumption of wine	Yes	34.4%
	No	65.6%
Consumption of Beer	Yes	40.6%
	No	59.4%
Consumption of Liquors	Yes	21.9%
	No	78.1%
**Variable**	**Median**	**Interquartile range**
Age	53	50–57
Adherence to Mediterranean diet	10	8–11
WHO score wellness	53.0%	42.0–72.0%
Body Mass index	25.8	24.3–28.8

**Table 3:** History of various diseases (n = 32).

Prior History of	Percentage
Heart attack	6.3%
Angina Pectoris	0
Stroke	0
High blood pressure	31.3%
High cholesterol or triglycerides	43.8%
Diabetes	3.1%
Gallstones	0
Kidney stones	9.4%
Intestinal polyps	0
Malignant tumors	6.3%
Gastric ulcer	0
Autoimmune diseases	21.9%

**Table 4:** Variation of blood tests after 14 days compared to baseline (n = 32).

Variable (Unit of Measure)	N Evaluable	At Baseline, Median	After 14 Days, Median	Median Difference (After 14 Days vs. Baseline)	*p* *
Hemoglobin (g/dL)	32	14.15	13.85	−0.3	0.0812
Heart Rate (bpm)	32	73.5	74	1.5	0.7151
**Fasting blood sugar (mg/dL)**	**32**	**82**	**74.5**	**−6**	**0.0001**
White Blood Cells (10^3^/µL)	32	6.86	6.595	−0.21	0.3171
Aspartate Aminotransferase U/L	32	20	17.5	−1.5	0.0931
Alanine Aminotransferase U/L	32	19.5	18.5	0	0.5003
**Reactive Oxygen Metabolites-D-ROMs (U.CARR)**	**32**	**394.5**	**365.5**	**−30**	**0.001**
Platelets 10^3^/µL	32	239	242	−2	0.6876
**Systolic Blood Pressure (mmHg)**	**31**	**133**	**122**	**−10**	**0.0001**
Diastolic Blood Pressure (mmHg)	31	84	81	−1	0.0528
Oxygen Saturation (%)	32	99	98	0	0.5843
Estimated Glomerular Filtration Rate (eGFR)	32	90	87	−4	0.1435

* Wilcoxon Signed-Rank. Statistically significant results are reported in bold.

## Data Availability

The data presented in this study are available upon request from the corresponding authors due to privacy safeguards.
